# Draft Genome Sequence of Phaeobacter sp. Strain JL2872, Isolated from Surface Waters in the South China Sea

**DOI:** 10.1128/MRA.00942-18

**Published:** 2018-10-11

**Authors:** Rong Cui, Yu Tian, Zhisheng Shao, Xiaopei Lin

**Affiliations:** a China National Offshore Oil Corporation Co., Ltd., Zhanjiang Branch, Zhanjiang, China; b State Key Laboratory of Marine Environmental Science, Institute of Marine Microbes and Ecospheres, Xiamen University, Xiamen, China; University of Arizona

## Abstract

Phaeobacter sp. strain JL2872 was isolated from surface waters in the South China Sea. We report here the draft genome sequence of strain JL2872, which can contribute to our understanding of the characteristics of the members of the genus Phaeobacter.

## Abstract (precis)

Phaeobacter sp. strain JL2872 was isolated from surface waters in the South China Sea.

## ANNOUNCEMENT

Phaeobacter sp. strain JL2872, which was isolated from surface waters (depth, 10 m) in the South China Sea, is a rod-shaped, yellow-pigmented, and Gram-negative bacterium. The phylogenetic analysis using 16S rRNA gene sequences showed that strain JL2872 formed a distinct lineage within the genus Phaeobacter and shared the highest sequence similarity with the type strain P. gallaeciensis BS107 (97.2%). P. gallaeciensis, a member of the abundant marine Roseobacter clade, is known to be an effective colonizer of biotic and abiotic marine surfaces. Its production of the antibiotic tropodithietic acid (TDA) makes P. gallaeciensis a strong antagonist of many bacteria, including fish and mollusk pathogens (1). Here, we report the draft genome sequence of Phaeobacter sp. strain JL2872.

In this project, genomic DNA was extracted and purified from cells cultivated in 2216E medium for 2 days at 22℃ using a Tguide bacterial genomic DNA kit (catalog number OSR-M502; Tiangen Biotech) following the manufacturer's instructions. Strain JL2872 was sequenced using paired-end reads on the MiSeq platform (Illumina, Inc., USA). A draft genome was assembled from these reads using the A5-miseq assembler (2). Coding sequences (CDSs) were predicted using GLIMMER version 3.0 (3). The total number of Illumina reads was 2,478,362, the total number of bases was 619,597,410, and the coverage was 134×. The draft genome of strain JL2872 contained 4,165,276 bp distributed in 26 DNA scaffolds, with a G+C content of 61.58%. The scaffold *N*_50_ value was 391,023 bp, the longest scaffold was 1,174,734 bp, and the shortest scaffold was 1,191 bp. The genome consisted of 53 tRNA genes, 3 rRNA operons, and 20 noncoding RNAs. We predicted the tRNA and rRNA genes using tRNAscan-SE version 1.31 (4) and RNAmmer version 1.2 (5), respectively.

Functional annotation was achieved using the Gene Ontology (GO), KEGG, Evolutionary Genealogy of Genes: Nonsupervised Orthologous Groups (eggNOG), and Nonredundant (NR) protein databases. GO annotation of protein-coding genes was performed using Blast2GO (6) software with default parameters. KEGG ortholog and pathway annotation of protein-coding genes is completed mainly by automatic annotation of the KEGG database using KASS version 2.0 (7). eggNOG annotation was performed by BLAST software using the eggNOG version 4 database. NR annotation used BLASTp version 2.2.30+ software. In the genome of JL2872, a carbon monoxide dehydrogenase was present. Phaeobacter sp. strain JL2872 was predicted to have a complete pentose-phosphate pathway. Genes for ammonium transporters and nitrate reductase were also present in the strain JL2872 genome.

### Data availability.

The shotgun genome sequence of Phaeobacter sp. strain JL2872 has been deposited at GenBank under the accession number QEOS00000000. The version described in this paper is the first version, QEOS01000000. The SRA accession number is SRP159216.
